# Natural commensal microbes induce internal hatching in *C. elegans*

**DOI:** 10.1128/spectrum.04034-25

**Published:** 2026-06-11

**Authors:** Nora M. Villafuerte, Emma N. Stevens, Mina A. Sheikh, Victoria H. Rodriguez, Jennifer Lin, Fan Zhang

**Affiliations:** 1 Department of Biological Sciences, Louisiana State University 124526, Baton Rouge, Louisiana, USA; 2 Department of Statistics, Rice University 224076, Houston, Texas, USA; Brigham Young University, Provo, Utah, USA

**Keywords:** *Caenorhabditis elegans *microbiome, insulin/IGF-1 signaling pathway, microbe–host interaction, facultative viviparity, reproductive plasticity

## Abstract

The microbiome is increasingly recognized as a key factor of the plasticity of host life-history traits. Using the *Caenorhabditis elegans* model system, we examined how naturally associated bacterial strains affect internal egg hatching, a stress-adaptive reproductive strategy in this species. Among the bacterial strains tested, four strains (*Ochrobactrum* BH3, *Lelliottia* JUb66, *Pantoea* BIGb0393, and *Enterobacter* CEent1) induced high levels of internal hatching (28–57%) in *C. elegans* during late adulthood, compared to <5% in animals grown on *E. coli* OP50. These effects were accompanied by extending the reproductive window while showing a trend toward reduced total progeny and lifespan. Genetic analyses revealed that these effects are mediated through various components of the insulin signaling pathway: the *α-proteobacterial* strain BH3 acts via DAF-16/FOXO, whereas the three γ*-proteobacterial* strains act independently of DAF-16, suggesting differences in pathway dependence. Our findings demonstrate that naturally associated microbiome members can differentially interact with host endocrine signaling to shape reproductive outcomes. These results underscore the role of microbial cues in shaping life-history plasticity in animals.

**IMPORTANCE:**

Microbiome members profoundly influence host physiology, including reproductive strategies. Using the *Caenorhabditis elegans* natural microbiome model, we show that commensal bacteria can induce internal egg hatching, a facultative vivipary phenotype previously linked primarily to early-life starvation or pathogen exposure that severely reduces reproductive output. In contrast, commensal strains trigger this shift mainly in late adulthood, extending the reproductive window with minimal impact on overall fecundity. We further demonstrate that bacterial strains act through distinct components of the host insulin signaling pathway. More broadly, these findings highlight diverse avenues within conserved endocrine networks that are susceptible to microbial modulation and underscore the potential to leverage microbiomes to influence host life-history traits.

## OBSERVATION

**Fig 1:**
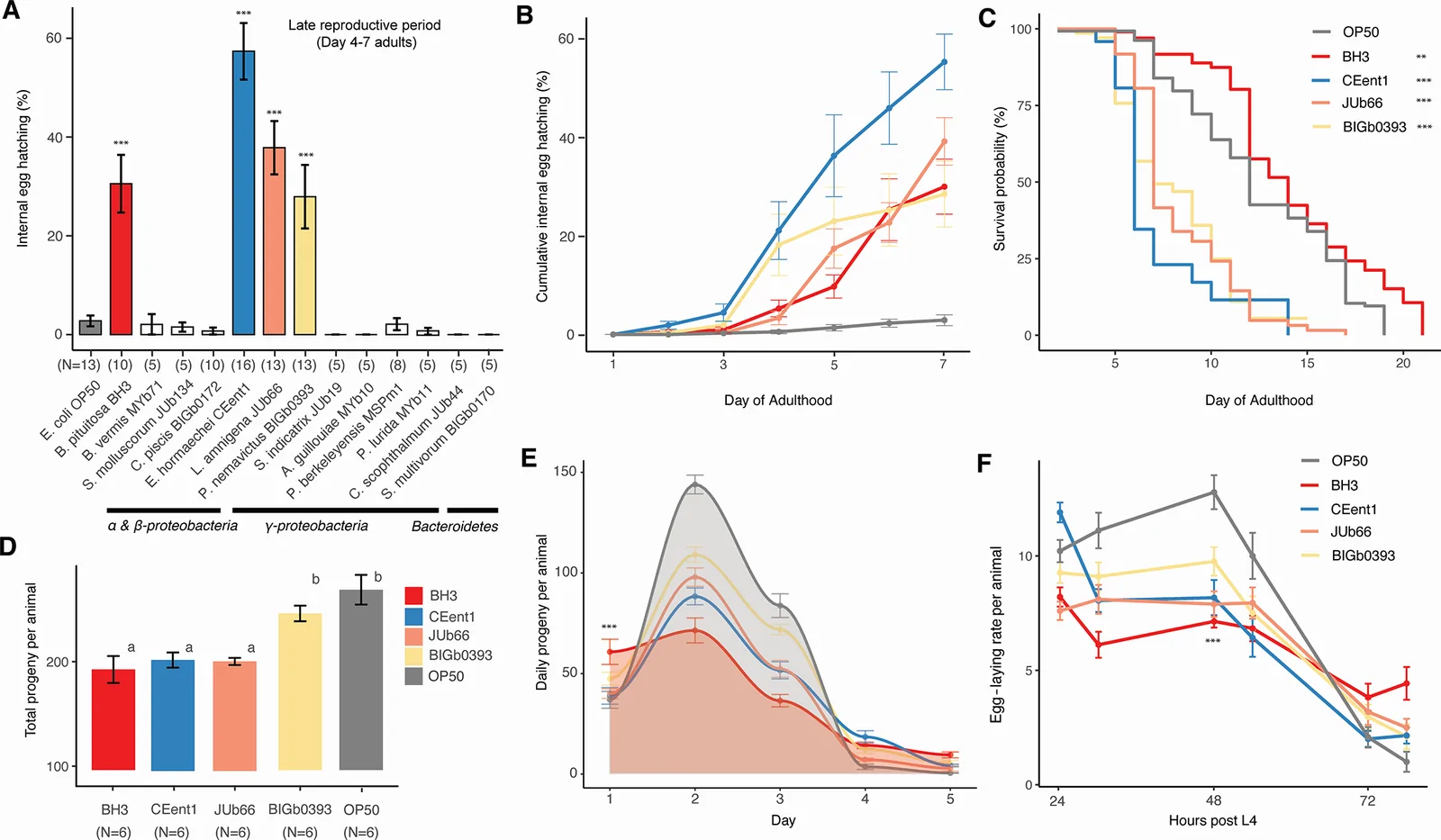
Natural commensal bacteria induce internal hatching and impact on lifespan and reproduction. (**A**) Mean percentage ± SE of *C. elegans* N2 that underwent internal egg hatching during late adulthood (days 4–7) when grown on 14 bacterial strains in monoculture on NGM plates at 20°C. Statistical significance was determined by comparison of natural bacterial strains to *E. coli* OP50 with a linear mixed model with Dunnett-adjusted post hoc comparisons (* adj *P* ≤ 0.05, ** adj *P* ≤ 0.01, *** adj *P* ≤ 0.001, *N* = 5–16 replicates per bacterial strain, *N* = 30 to 32 individual animals per replicate; see Table S1 for details). (**B**) Cumulative daily percentage of worms that underwent internal hatching over adulthood. Points represent mean ± SE across biological replicates (*N* = 10–16 replicates per bacterial strain from two independent experiments, *N* = 30 to 32 individuals per replicate; see Table S2 for details). (**C**) Kaplan–Meier survival curves showing daily survival probabilities of *C. elegans* adults. Internal hatching events were scored as censored worms and removed from survival calculations (*N* = 5 replicates per bacterial strain, *N* = 30 individuals per replicate; see Table S3 for details). (**D**) Total number of progeny produced per worm (mean ± SE) over the reproductive period. Different letters above bars indicate statistically significant differences among bacterial treatments (one-way ANOVA followed by Tukey’s HSD post hoc test, *P* < 0.05, *N* = 6 plate replicates for each bacterial strain; see Table S4 for details). (**E**) Smoothed curves show the daily progeny produced per worm (mean ± SE) over the reproductive period. Significance on day 1 adulthood is indicated for BH3 versus OP50. (*N* = 6 plate replicates for each bacterial strain; see Table S5 for details). (**F**) Hourly egg-laying rates (mean ± SE) of adults grown on focal bacterial strains. Significance at 48 h post-L4 is indicated for four focal strains versus OP50. Data are compiled from two independent experiments (*N* = 14–30 individuals per time point per bacterial strain; see Table S6 for details).

The free-living nematode *Caenorhabditis elegans* typically lays eggs that hatch externally, allowing larvae immediate access to environmental nutrients. However, worms may retain eggs under stress, leading to larvae hatching within the body that often results in premature maternal death, a phenomenon first described in early studies of egg-laying behavior in the 1980s (1, 2). This process, termed facultative viviparity, refers to the retention and internal hatching of embryos within the parent under specific physiological or environmental conditions. Facultative viviparity represents a plastic life-history trait that balances maternal survival and offspring fitness (2, 3), and may function as a presumptive adaptive strategy that enhances survival under stressful conditions in adulthood (4), analogous to dauer formation during development. The transition between oviparity and viviparity in *C. elegans* is regulated by the interplay of genetic and environmental factors. For example, the insulin receptor mutant, which reduces nutrient-sensing pathway insulin signaling, shows an increase in internal hatching (5). Disruption of serotonin signaling can delay egg laying and promote internal hatching, and natural variation in the calcium-activated potassium channel gene *kcnl-1* contributes to this phenotype in wild populations (4, 6). Environmental factors such as nutrient limitation strongly induce internal hatching, while oxygen deprivation and elevated temperature can produce similar effects (2, 7, 8). In addition, exposure to animal pathogens increases internal hatching (9). In nature, *C. elegans* encounters diverse microbes that serve as food sources and commensal partners (10–13). However, their influence on reproductive mode is not fully characterized. Here, we use the *C. elegans* microbiome resource to assess their impact on internal hatching. We tested 12 strains from the CeMbio collection (14). In addition, *Ochrobactrum pituitosa* (BH3), a naturally associated strain isolated from wild *C. elegans* and a member of the BIGbiome community, was included based on a previous study linking its colonization to host insulin signaling (15) and to compare with the CeMbio *Ochrobactrum* strain MYb71 from the same genus. The standard diet *E. coli* OP50 was used as the baseline benchmark. Wild-type N2 worms fed OP50 from the L1 stage showed minimal internal hatching (0.29% ± 0.29% early; 2.77% ± 1.10% late adulthood). None of the natural strains increased internal hatching during early adulthood (Fig. S1), contrasting with the ~59% early-adulthood induction reported for pathogens (9). However, four strains significantly elevated it during late reproduction: *O. pituitosa* BH3 (30.6% ± 5.8%), *Lelliottia amnigena* JUb66 (37.8% ± 5.4%), *Pantoea nemavictus* BIGb0393 (27.9% ± 6.4%), and *Enterobacter hormaechei* CEent1 (57.4% ± 5.7%) (Fig. 1A and B).

We next examined the effects of the four focal bacterial strains on life-history traits, including lifespan, reproduction, and egg-laying rates. Worms grown on BH3 showed an extended mean lifespan (14.2 ± 0.47 days) compared to OP50 (12.5 ± 0.37 days, *P* < 0.01). In contrast, the other focal strains significantly shortened lifespan: BIGb0393 (8.5 ± 0.59 days), CEent1 (7.2 ± 0.66 days), and JUb66 (8.3 ± 0.30 days, *P* < 0.0001) (Fig. 1C). Total progeny per animal was approximately 20% lower in three of the four strains—BH3 (192.6 ± 12.9), CEent1 (201.6 ± 7.3), and JUb66 (200.3 ± 3.4)—compared to OP50 (268.8 ± 14.2). BIGb0393 (246.3 ± 7.5) showed a smaller reduction (Fig. 1D). Daily reproductive output revealed altered dynamics: N2 worms grown on BH3 produced more offspring on day 1 (adj *P* < 0.0001), showed reduced output during the typical reproductive peak (days 2–3), and maintained slightly higher output later (days 4–5), resulting in a flattened reproductive curve relative to OP50 (Fig. 1E). This extended reproductive window is generally associated with reduced total fecundity and lifespan. BH3 represents an exception, as it enhances both lifespan and the duration of the reproductive window. Hourly egg-laying analyses showed that worms on focal strains had lower egg-laying rates during the peak reproductive period (adj *P* < 0.0001) compared to OP50 (Fig. 1F), thereby potentially increasing egg retention and promoting internal hatching.

**Fig 2:**
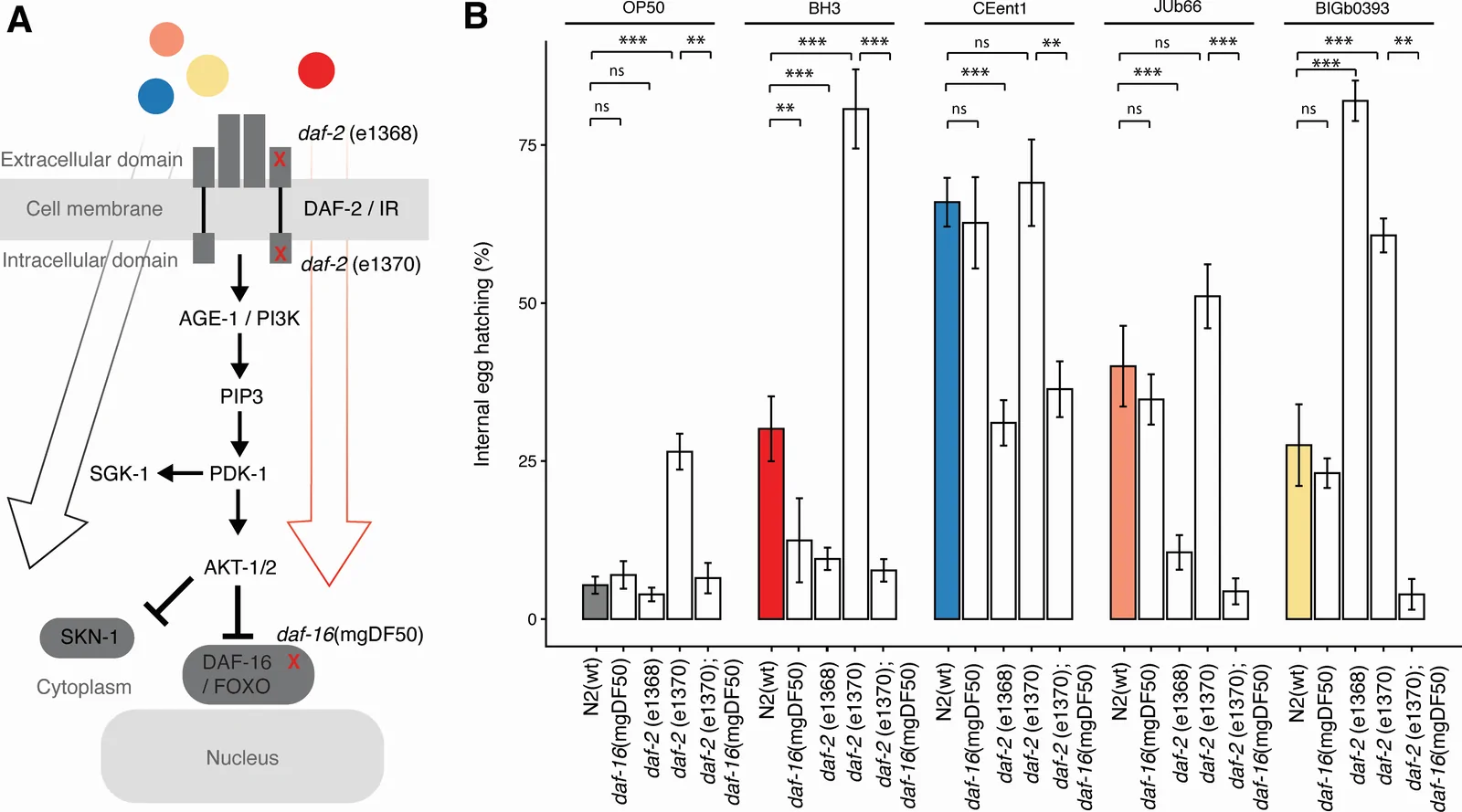
Interactions of focal strains with host insulin signaling. (**A**) Schematic of the *C. elegans* insulin/IGF-1 signaling pathway and its modulation by focal bacterial strains. Core components include DAF-2, AGE-1/PI3K, PDK-1, AKT-1/2, SGK-1, and the transcription factors DAF-16/FOXO and SKN-1/NRF2. Colored circles in the extracellular space represent bacterial-derived metabolites or signals from four focal strains. Loss-of-function mutation alleles (*daf-2(e1368*), *daf-2(e1370*), *daf-16(mgDf50*)) are indicated at their respective pathway positions. Shaded arrows denote proposed pathway branches influenced by bacterial strains based on genetic dependence observed in this study. (**B**) Internal egg hatching percentages of *C. elegans* insulin signaling mutants (*daf-16(mgDf50*), *daf-2(e1368*), *daf-2(e1370*), and *daf-2(e1370);daf-16(mgDf50*) double mutants) were quantified after exposure to focal bacterial strains. Data are shown as percentage of worms with internal hatching (mean ± SE, ns adj *P* > 0.05, * adj *P* ≤ 0.05, ** adj *P* ≤ 0.01, *** adj *P* ≤ 0.001, *N* = 11–16 replicates per bacterial strain from two independent trials, *N* = 25 to 30 individual animals per replicate). Exact statistical results are provided in Tables S7 to S9 (S7: summary statistics; S8: Dunnett-adjusted comparisons of bacterial effects within genotype; and S9: Dunnett-adjusted comparisons of genotype effects within bacterial condition).

We then asked whether the elevated internal egg hatching observed on the focal bacterial strains depends on the insulin signaling pathway, a key regulator of reproductive and stress responses in *C. elegans*. Under stress, reduced insulin-like signaling promotes nuclear localization of the transcription factor DAF-16, activating stress-resistance and reproduction-related programs that can slow egg laying and increase internal hatching (16). To test whether bacterial effects are mediated through this pathway, we analyzed four *C. elegans* mutants with loss-of-function mutations in the insulin receptor and downstream transcription factors (Fig. 2A). Overall, a significant bacteria × genotype interaction (likelihood ratio test, χ² = 595.8, df = 16, *P* = 1.81 × 10⁻¹⁶) indicates that the effects of bacterial strains on internal hatching differ across genotypes. Specifically, *daf-16(mgDf50*) loss-of-function mutants grown on BH3 showed a marked reduction in internal hatching (12.4% ± 6.6%) compared to N2 wild type (30.1% ± 5.1%), while no significant changes were observed for CEent1, JUb66, or BIGb0393 (Fig. 2B). These results indicate that BH3-induced internal hatching acts in a DAF-16-dependent manner. In contrast, the three γ-proteobacterial strains appear to act independently of DAF-16, suggesting the involvement of other branches of DAF-2 signaling pathways, such as SGK-1 or SKN-1 (17, 18) (Fig. 2A), or possibly through serotonin-mediated egg-laying circuitry.

To further determine the role of the insulin receptor, we analyzed *daf-2* mutants. The class 1 allele *daf-2(e1368*) carries a mutation in the extracellular ligand-binding domain and exhibits normal egg-laying on OP50 (19). Exposure to focal bacterial strains increased internal hatching relative to OP50 but to a lesser extent than in wild type, suggesting that an intact DAF-2 receptor is required for full responsiveness (Fig. 2B; Fig. S2). The exception was BIGb0393, which induced a dramatic increase in internal hatching (82.0% ± 3.2% vs. 27.5% ± 6.5% in N2, adj. *P* < 0.0001), implying that its signals may interact with the altered ligand-binding pocket to enhance receptor activation. The class 2 allele *daf-2(e1370*), carrying a mutation in the intracellular kinase domain that disrupts PI3-kinase signaling, constitutively increases internal hatching even on OP50 (19). In this background, internal hatching was further elevated across most focal strains (Fig. 2B; Fig. S3), with the highest levels observed for BH3 (80.7% ± 6.3%), BIGb0393 (60.7% ± 2.9%), and JUb66 (51.1% ± 5.1%). Only CEent1 showed no additional increase, possibly reflecting a ceiling effect where internal hatching had already reached a maximal level. The *daf-2(e1370)*;*daf-16(mgDf50*) double mutant exhibited a strong reduction in internal hatching across all strains (Fig. 2B; Fig. S2), supporting the central role of DAF-16 in mediating this phenotype. For JUb66 and BIGb0393, internal hatching dropped to near-baseline levels, suggesting that their effects are reprogrammed through DAF-16 under reduced insulin signaling.

Together, these results support a model in which bacteria-derived cues modulate internal hatching through differential dependence on conserved insulin signaling components. Future work should examine how bacterial cues influence insulin ligand expression and explore additional regulators, including SGK-1 and SKN-1, in mediating bacteria-induced internal hatching. Importantly, our findings reflect pathway dependence inferred from genetic perturbations and do not constitute direct measurements of signaling activity. Direct readouts of pathway activity, such as transcriptional or subcellular localization reporters for DAF-16 (20, 21), will be essential to determine how microbial signals are integrated within IIS to regulate reproductive outcomes. In addition, given the dynamic nature of host–microbe interactions across the lifespan (22), bacterial effects may shift over time. Although these strains support accelerated development relative to OP50 (14) and robust reproduction, reduced nutritional quality or mild pathogenic effects may emerge later in adulthood after day 4. Direct metabolic readouts such as lipid staining (23) during late adulthood will be needed to determine whether nutritional effects contribute to internal hatching observed. Finally, testing defined bacterial consortia will help to elucidate how microbiome composition fine-tunes reproductive strategies in *C. elegans* in their natural environment.

### Worm maintenance

*C. elegans* strains were obtained from the Caenorhabditis Genetics Center: N2 (wild type), CB1368 [*daf-2*(e1368)], CB1370 [*daf-2*(e1370)], GR1307 [*daf-16*(mgDf50)], and HT1890 [*daf-2*(e1370);*daf-16*(mgDf50)]. Worms were maintained under standard laboratory conditions at 20**°**C until adulthood. Gravid adults were treated with alkaline bleach solution following standard procedures to isolate eggs, which were then incubated overnight in M9 buffer on a rotator at room temperature to allow hatching. The resulting larvae in developmental stage 1 (L1) were age-synchronized and subsequently seeded onto nematode growth medium (NGM) plates containing the bacterial strain of interest.

### Bacterial preparation

Each bacterial strain was inoculated from a single colony into lysogeny broth (LB) and cultured overnight at 30°C with shaking at 120 rpm. Before seeding onto NGM plates, the optical density (OD₆₀₀) of each culture was measured using a BioTek Epoch 2 microplate reader and adjusted to OD = 1.0 for equal bacterial density across conditions.

### Scoring of internal egg hatching and lifespan assay

During the reproductive period of adulthood, worms were transferred daily to fresh NGM plates using a sterilized platinum worm pick under a stereo microscope (Nikon SMZ-745). Each worm was scored as alive, dead, internal hatched, or censored (lost or desiccated individuals). Internal egg hatching was identified by the presence of retained larvae within the adult body. Internal egg hatching percentage was calculated as the number of individuals with internal hatching divided by the cohort size, excluding censored worms. In lifespan assays, worms exhibiting internal egg hatching were treated as censored and excluded in the survival curve. For each bacterial condition, 30 adult worms per plate were used, with >150 worms per condition scored across replicates.

### Reproduction and egg-laying assay

Three L4-stage hermaphrodites were transferred daily to fresh plates until the end of reproduction (day 5). Progeny on vacated plates were counted 48 h later to include all hatched offspring, and the total brood size per worm was calculated as the sum of progeny across days. Each condition included at least six plates. Worms exhibiting internal hatching were excluded from further reproductive scoring. For the egg-laying assay, single adult hermaphrodites were placed on fresh bacterial lawns for 2 hours each day, after which the eggs laid were counted immediately under a stereomicroscope. Hourly egg-laying rate per animal was determined daily from day 1 to day 4 of adulthood, with a minimum of 30 individuals analyzed per condition.

### Statistical analysis

All analyses were performed in R (v4.3.0) using dplyr, ggplot2, lme4*,* and survival packages. Internal hatching percentages were calculated as the proportion of worms exhibiting internal hatching relative to the total number of worms per plate and are presented as mean ± SE. These data were modeled as a binomial response using generalized linear mixed-effects models (GLMMs) implemented in the *lme4* package, with bacterial strain and genotype included as fixed effects and biological replicate (plate) included as a random effect to account for non-independence within plates. Post hoc comparisons between bacterial strains and the OP50 control were performed using estimated marginal means with Dunnett-adjusted contrasts in the *emmeans* package. The significance of the bacteria × genotype interaction was assessed using likelihood ratio tests by comparing models with and without the interaction term. Total reproductive output was analyzed using one-way ANOVA, followed by Tukey’s HSD post hoc test. Daily reproductive output and egg-laying rates were analyzed using mixed-effects models with bacterial strain, day, and their interaction as fixed effects, and biological replicate included as a random effect. Post hoc comparisons were performed using estimated marginal means with Dunnett-adjusted contrasts. Lifespan data were analyzed using Kaplan–Meier survival curves generated from daily census data, and internal hatching egg events were not treated as death events in the survival model. Survival differences among bacterial conditions were assessed using log-rank (Mantel–Cox) tests with Benjamini–Hochberg correction applied for multiple comparisons.

## Supplementary Material

Reviewer comments

## Data Availability

All data supporting the findings of this study are included in the supplemental tables. Strains from the CeMbio collection and *C. elegans* genetic mutants used in this study are available from the Caenorhabditis Genetics Center (CGC) or upon request from the corresponding author.

## SUPPLEMENTAL MATERIAL

Supplemental Figures spectrum.04034-25-s0001.docx Fig. S1 and S2.

Supplemental Tables spectrum.04034-25-s0002.xlsx Tables S1 to S9.

OPEN PEER REVIEW reviewer-comments.pdf An accounting of the reviewer comments and feedback.

ASM does not own the copyrights to Supplemental Material that may be linked to, or accessed through, an article. The authors have granted ASM a non-exclusive, world-wide license to publish the Supplemental Material files. Please contact the corresponding author directly for reuse.
