# Supplementary material for: Natural commensal microbes induce internal hatching in C. elegans
Source: Microbiol Spectr. 2026 Jun 11;14(7):e04034-25. doi: 10.1128/spectrum.04034-25 (PMC13340241; doi:10.1128/spectrum.04034-25)
Supplement: Supplemental Figures — Fig. S1 and S2. [file spectrum.04034-25-s0001.docx]

**Supplementary Materials**

**
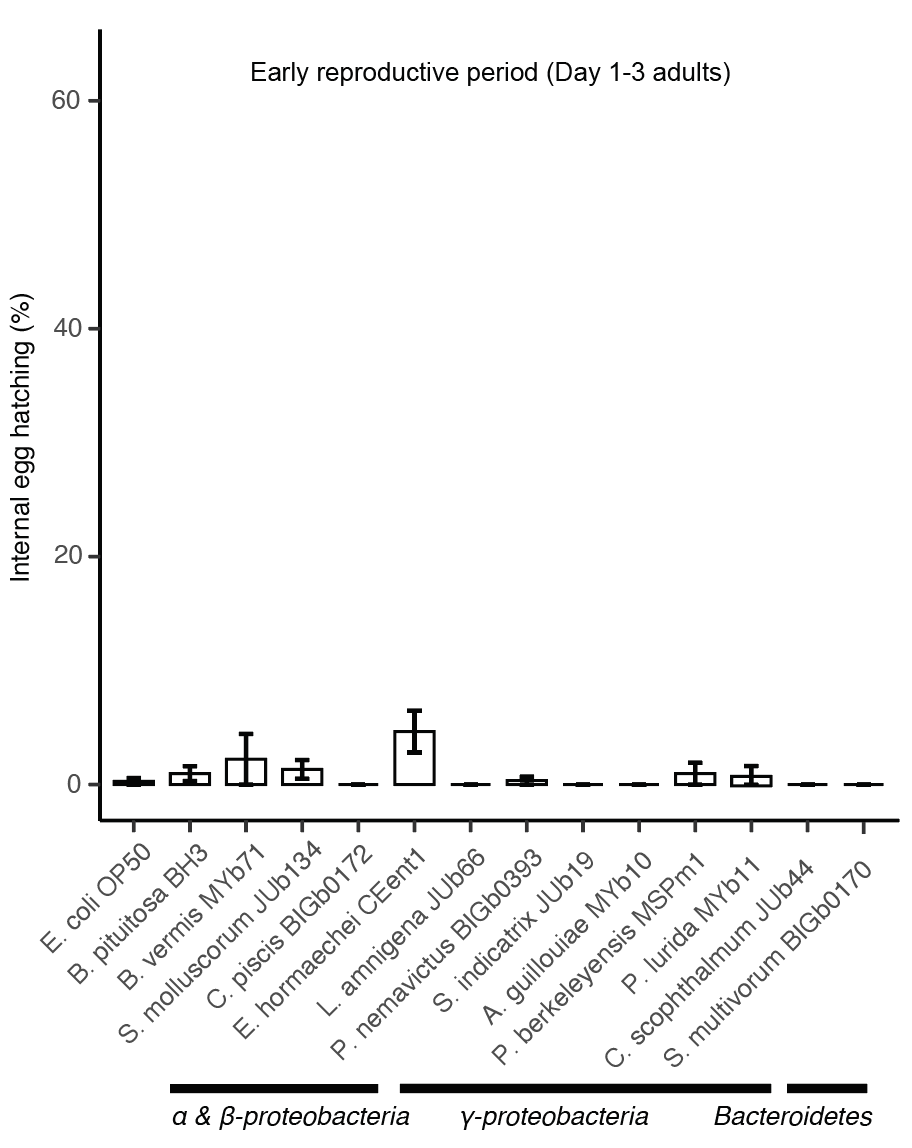
**

**Figure S1. Impact of natural commensal bacteria on internal hatching during early adulthood.** Bar plots show the mean percentage (± SE) of worms that underwent internal egg hatching early (Days 1–3) adulthood stage for 14 bacterial strains monoculture on NGM plates at 20 °C.


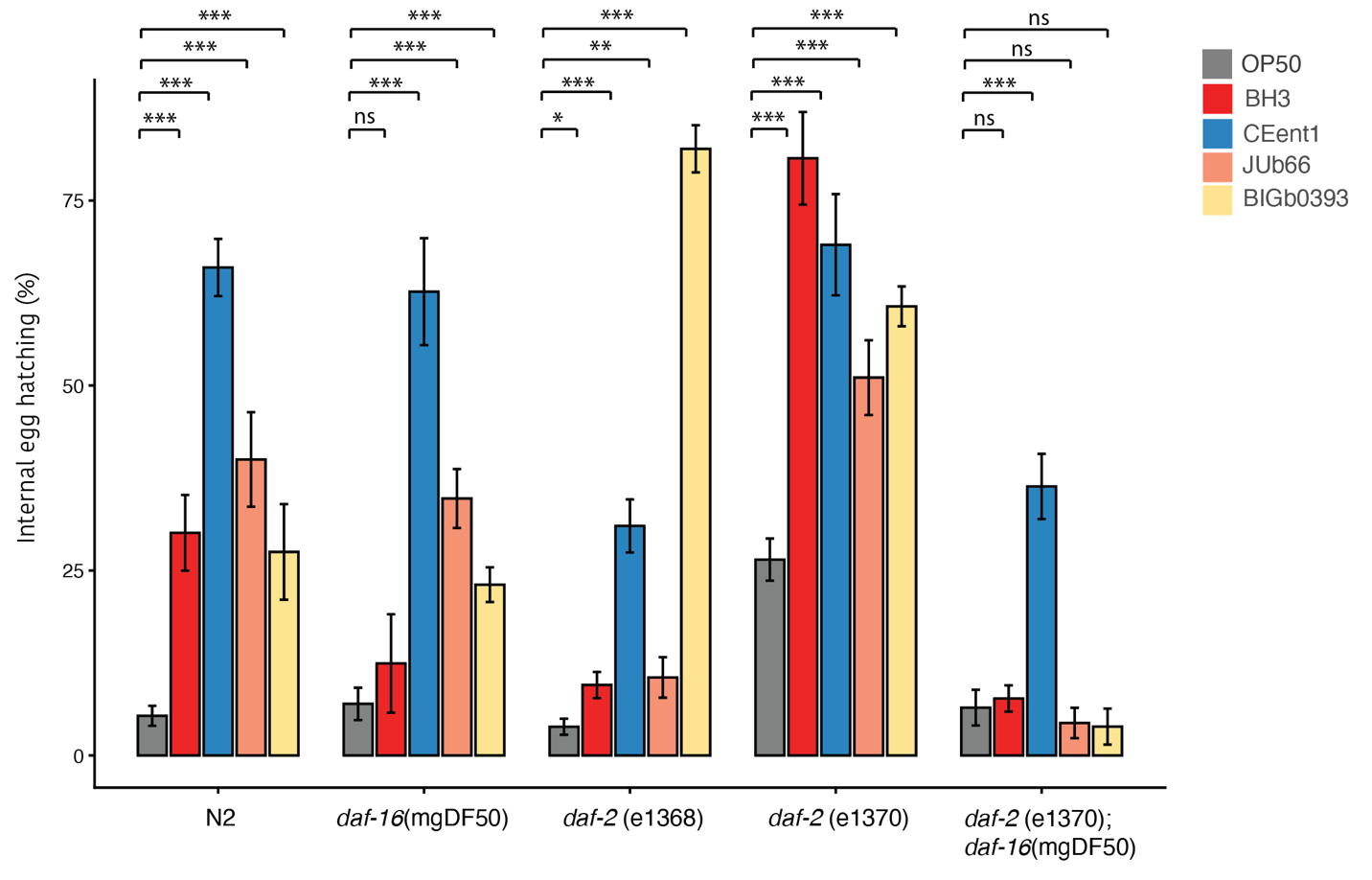


**Figure S2.**  **Internal hatching percentages of across bacterial conditions grouped by *C. elegans* genotype**: N2, *daf-16*(mgDf50), *daf-2*(e1368), *daf-2*(e1370), and *daf-2*(e1370); *daf-16*(mgDf50). Data are shown as percentage of worms with internal hatching (mean ± SE). Statistical analyses were performed using GLMMs with Dunnett-adjusted comparisons versus OP50 within each genotype. Exact statistics are provided in Supplementary Table S8.
