# Supplementary material for: Natural commensal microbes induce internal hatching in C. elegans
Source: Microbiol Spectr. 2026 Jun 11;14(7):e04034-25. doi: 10.1128/spectrum.04034-25 (PMC13340241; doi:10.1128/spectrum.04034-25)
Supplement: Reviewer comments [file reviewer-comments.pdf]

AMERICAN SOCIETY FOR MICROBIOLOGY | Microbiology Spectrum

Natural commensals induce internal egg hatching in *C. elegans*

Nora Villafuerte, Emma Stevens, Mina Sheikh, Jennifer Lin, Victoria Rodriguez, and Fan Zhang

Corresponding Author(s): Fan Zhang, Louisiana State University Department of Biological Sciences

Review Timeline:

Submission Date:	December 15, 2025
Editorial Decision:	December 16, 2025
Revision Received:	December 16, 2025
Editorial Decision:	January 26, 2026
Revision Received:	March 26, 2026
Accepted:	May 16, 2026

Editor: John Chaston

Reviewer(s): The reviewers have opted to remain anonymous.

Transaction Report:

(Note: With the exception of the correction of typographical or spelling errors that could be a source of ambiguity, letters and reports are not edited. The original formatting of letters and referee reports may not be reflected in this compilation.)

DOI: https://doi.org/10.1128/spectrum.04034-25

Re: Spectrum04034-25 (**Natural commensals induce internal egg hatching in *C. elegans***)

Dear Dr. Fan Zhang:

Thank you for your help sending me the files earlier today, but the only way to modify is to send back. So, I'm sending back, with details below on resubmitting. Hopefully we get it through QC and off for review this week. Sorry for the confusion, I had thought trying the other way would help.

Please return the manuscript within 60 days; if you cannot complete the modification within this time period, please contact me. If you do not wish to modify the manuscript and prefer to submit it to another journal, notify me immediately so that the manuscript may be formally withdrawn from consideration by Spectrum.

Revision Guidelines
To submit your modified manuscript, log into the submission site at https://spectrum.msubmit.net/cgi-bin/main.plex. Go to Author Tasks and click the appropriate manuscript title to begin. The information you entered when you first submitted the paper will be displayed; update this as necessary. Note the following requirements:

• Upload point-by-point responses to the issues raised by the reviewers in a file named "Response to Reviewers," NOT in your cover letter.
• Upload a compare copy of the manuscript (without figures) as a "Marked-Up Manuscript" file.
• Upload a clean .DOC/.DOCX version of the revised manuscript and remove the previous version.
• Each figure must be uploaded as a separate, editable, high-resolution file (TIFF or EPS preferred), and any multipanel figures must be assembled into one file.
• Any supplemental material intended for posting by ASM should be uploaded with their legends separate from the main manuscript. You can combine all supplemental material into one file (preferred) or split it into a maximum of 10 files with all associated legends included.

For complete guidelines on revision requirements, see our Submission and Review Process webpage. Submission of a paper that does not conform to guidelines may delay acceptance of your manuscript.

Data availability: ASM policy requires that data be available to the public upon online posting of the article, so please verify all links to sequence records, if present, and make sure that each number retrieves the full record of the data. If a new accession number is not linked or a link is broken, provide Spectrum production staff with the correct URL for the record. If the accession numbers for new data are not publicly accessible before the expected online posting of the article, publication may be delayed; please contact production staff (Spectrum@asmusa.org) immediately with the expected release date.

Publication Fees: For information on publication fees and which article types are subject to charges, visit our website. If your manuscript is accepted for publication and any fees apply, you will be contacted separately about payment during the production process; please follow the instructions in that e-mail. Arrangements for payment must be made before your article is published.

ASM Membership: Corresponding authors may join or renew ASM membership to obtain discounts on publication fees. Need to upgrade your membership level? Please contact Customer Service at Service@asmusa.org.

The ASM Journals program strives for constant improvement in our submission and publication process. Please tell us how we can improve your experience by taking this quick Author Survey.

Thank you for submitting your paper to Spectrum.

Sincerely,
John Chaston
Editor
Microbiology Spectrum

Re: Spectrum04034-25R1 (**Natural commensals induce internal egg hatching in *C. elegans***)

Dear Dr. Fan Zhang:

Thank you for the privilege of reviewing your work. Below you will find my comments, instructions from the Spectrum editorial office, and the reviewer comments.

I have received the reviews of your manuscript and regret to inform you that at this time the current version of the manuscript has issues that, if unaddressed, would not be suitable for publication in Microbiology Spectrum. Your submission was read by two reviewers with expertise in the area addressed in your study and both raised issues about the statistical transparency and rigor, as well as other issues. If you are able to address these issues I would welcome a revised version, but that resubmission would require a new round of review upon which the reviewers would be able to identify and raise new issues whether in response to your revisions or not. If you choose to resubmit, please follow the instructions below.

Please return the manuscript within 60 days; if you cannot complete the modification within this time period, please contact me. If you do not wish to modify the manuscript and prefer to submit it to another journal, notify me immediately so that the manuscript may be formally withdrawn from consideration by Spectrum.

Revision Guidelines
To submit your modified manuscript, log into the submission site at https://spectrum.msubmit.net/cgi-bin/main.plex. Go to Author Tasks and click the appropriate manuscript title to begin. The information you entered when you first submitted the paper will be displayed; update this as necessary. Note the following requirements:

• Upload point-by-point responses to the issues raised by the reviewers in a file named "Response to Reviewers," NOT in your cover letter.
• Upload a compare copy of the manuscript (without figures) as a "Marked-Up Manuscript" file.
• Upload a clean .DOC/.DOCX version of the revised manuscript and remove the previous version.
• Each figure must be uploaded as a separate, editable, high-resolution file (TIFF or EPS preferred), and any multipanel figures must be assembled into one file.
• Any supplemental material intended for posting by ASM should be uploaded with their legends separate from the main manuscript. You can combine all supplemental material into one file (preferred) or split it into a maximum of 10 files with all associated legends included.

For complete guidelines on revision requirements, see our Submission and Review Process webpage. Submission of a paper that does not conform to guidelines may delay acceptance of your manuscript.

Data availability: ASM policy requires that data be available to the public upon online posting of the article, so please verify all links to sequence records, if present, and make sure that each number retrieves the full record of the data. If a new accession number is not linked or a link is broken, provide Spectrum production staff with the correct URL for the record. If the accession numbers for new data are not publicly accessible before the expected online posting of the article, publication may be delayed; please contact production staff (Spectrum@asmusa.org) immediately with the expected release date.

Publication Fees: For information on publication fees and which article types are subject to charges, visit our website. If your manuscript is accepted for publication and any fees apply, you will be contacted separately about payment during the production process; please follow the instructions in that e-mail. Arrangements for payment must be made before your article is published.

ASM Membership: Corresponding authors may join or renew ASM membership to obtain discounts on publication fees. Need to upgrade your membership level? Please contact Customer Service at Service@asmusa.org.

The ASM Journals program strives for constant improvement in our submission and publication process. Please tell us how we can improve your experience by taking this quick Author Survey.

Thank you for submitting your paper to Spectrum.

Sincerely,
John Chaston
Editor
Microbiology Spectrum
Reviewer #1 (Comments for the author):

The manuscript by Villafuerte et al. investigates how naturally associated bacteria modulate facultative vivipary (internal egg hatching) in Caenorhabditis elegans. By leveraging the CeMbio microbiome resource together with well-established insulin/IGF-

1 signaling mutants, the authors demonstrate that distinct commensal bacterial taxa induce internal hatching predominantly in late adulthood and engage different branches of the host insulin signaling network. The study is timely and addresses an important gap in our understanding of how natural microbiome members shape host reproductive strategies beyond pathogenic or starvation contexts.
Overall, the work is well motivated, the experimental approaches are technically sound, and the conclusions are largely supported by the presented data. The integration of life-history traits with genetic dissection of insulin signaling is a particular strength. Nevertheless, several aspects of data presentation, clarity of interpretation, and mechanistic framing require further refinement to improve rigor and accessibility. Addressing these points would substantially strengthen the manuscript without necessitating major additional experimentation.

Minor Comments
Comment 1. Statistical reporting
Please include exact p-values and sample sizes (number of worms analyzed) for all quantitative analyses, either in the main text, figure legends, or supplementary tables. In addition, a schematic model summarizing the proposed strain-specific engagement of insulin signaling branches would help readers grasp the key mechanistic conclusions.
Comment 2. Terminology clarification
Briefly define "facultative vivipary" at its first occurrence for readers who may not be specialists in C. elegans reproductive biology.
Comment 3. Figure organization
For improved clarity and conciseness, consider combining Figure 2A and 2B into a single panel, and grouping Figure 2C, 2D, and 2E into a unified multi-panel figure with consistent formatting.
Comment 4. Reference needed
Line 43: "leading to larvae hatching within the body that often results in premature maternal death."
Please provide an appropriate reference supporting this statement, as it is a key biological premise.
Comment 5. Text clarity
Line 18: "The three γ-proteobacterial strains belong to γ-proteobacteria"
This sentence is redundant and grammatically incorrect. Please revise for clarity and precision.
Comment 6. Reference needed
Line 61: "with the ~59% early adulthood induction reported for pathogens."
Please cite the original study or studies reporting this level of pathogen-induced internal hatching in early adulthood.

Reviewer #2 (Comments for the Author):

This manuscript investigates the influence of a number of naturally co-occurring bacteria (mainly from the CeMbio resource) on internal hatching in the nematode C. elegans. The study finds that some bacterial strains induce internal hatching preferentially in late adulthood, similar to what has been found for some pathogenic bacteria. The authors also use daf-2 and daf-16 mutants to make inferences about the involvement of insulin signalling.
The manuscript has conceptual and statistical weaknesses that limit the strength and reliability of the conclusions. Overall, the study feels very preliminary and the results should be treated/interpreted accordingly.

Main points
1. Interpretation of daf-2 signalling
The authors conclude that different bacterial strains engage distinct components of the insulin signaling pathway, including "canonical" and "non-canonical" signaling. This conclusion is based exclusively on phenotypic analysis of daf-2 and daf-16 mutants. No additional evidence is provided, e.g. measurements of pathway activity, which are relatively easy to obtain using available reporters. Overall, the data demonstrate genetic dependence, but not pathway engagement.

2. Nutrient effects of bacteria
The bacteria exert their effects also / primarily as nutrition. This aspect is not or insufficiently discussed. The simplest interpretation is that bacteria inducing internal hatching represent low-quality or mildly pathogenic food sources (as further suggested by the reproductive output assays). It would have been interesting to test this specifically, e.g. by assessing whether bacteria that induce internal hatching also induce mild starvation. For example, see the recent paper, which used lipid stains to look at that.
Braun, N. J., and D. E. Mor (2025). Gut microbiome species Levilactobacillus brevis regulates reproductive fitness in C. elegans. Curr. Res. Microb. Sci. 9: 100471.
https://pubmed.ncbi.nlm.nih.gov/41018064/

3. Experimental design
The experimental design and statistical analyses are of insufficient quality. It is unclear whether all experiments that were compared together were actually performed in parallel. More importantly, it is unclear how ANOVAs were performed-no detailed statistical test results (degrees of freedom, F values, etc.) are provided. Apparently, plate/replicate effects were not taken into account, which is problematic/incorrect. Similarly, figures do not display such replicate variation. Crucially, bacteria were all

pairwise compared to OP50, apparently without correcting for multiple comparisons. This is not transparent.
Figure 2: It is unclear which comparisons between genotypes/treatments were statistically tested and how.

Other comments
Abstract: It is unclear what the reported percentages refer to: "significantly increased the incidence of internal hatching (28-57%) during late adulthood."
Percent of individuals? Percent of populations? Increase relative to what baseline (0%, OP50, early adulthood)?
Lines 44-45:
"This facultative vivipary represents a plastic life-history trait that balances maternal survival and offspring fitness."
Comment: Reference 1 seems inappropriate here. Also, the term "stress-adaptive reproductive strategy" is unusual. Perhaps "a presumptive adaptive strategy that enhances survival under stressful conditions."
Line 58: "OP50-fed worms"
Comment: Specify nematode genetic background (presumably wild-type N2). Also specify developmental stage at feeding (L1/egg or L4). Although mentioned elsewhere, it would be helpful to state it here.
Line 57: Ochrobactrum pituitosa (BH3)
Comment: Why was this species chosen?
Line 61: Missing citation.
Lines 75-77:
"This extended reproductive window occurs at the expense of total fecundity and lifespan."
Comment: This should be reworded to avoid unwarranted causal interpretation. Why must this represent a trade-off?

Villafuerte Microbiology Spectrum 2026 Reviewer's Comments

Dear Editor,

We thank you and the reviewers for the careful evaluation of our manuscript. The comments and suggestions were very valuable and we have used them to further improve our manuscript. Below, we explain in more detail how exactly we responded to the comments, and have added additional experiments and analyses along with improvements to the text.

Please let us know if there are any specific questions!

Warmest regards,

Fan Zhang

Detailed response to reviewers' comments

(Please note that reviewers comments are given in *gray and italics*, while our response is given in blue and changes to the text are in **black with line number based on track change version**.)

Editor

I have received the reviews of your manuscript and regret to inform you that at this time the current version of the manuscript has issues that, if unaddressed, would not be suitable for publication in Microbiology Spectrum. Your submission was read by two reviewers with expertise in the area addressed in your study and both raised issues about the statistical transparency and rigor, as well as other issues. If you are able to address these issues I would welcome a revised version, but that resubmission would require a new round of review upon which the reviewers would be able to identify and raise new issues whether in response to your revisions or not. If you choose to resubmit, please follow the instructions below.

Please return the manuscript within 60 days; if you cannot complete the modification within this time period, please contact me. If you do not wish to modify the manuscript and prefer to submit it to another journal, notify me immediately so that the manuscript may be formally withdrawn from consideration by Spectrum.

Response: We thank you for the careful evaluation of our manuscript and for providing us the opportunity to revise and resubmit. We also appreciate the detailed and constructive feedback from the reviewers.

In response, we have undertaken substantial revisions to address these concerns. Specifically, we have implemented a generalized linear mixed-effects modeling framework to appropriately account for biological replicate (plate) effects, and have incorporated multiple-comparison corrections using Dunnett-adjusted contrasts. We have also expanded statistical reporting throughout the manuscript, including detailed test statistics, sample sizes, and adjusted p-values provided in revised figure legends and new supplementary tables (S1-9). In addition, we reorganized the panel structure of Figure 2 and included a schematic model summarizing strain-specific effects on insulin signaling pathway to illustrate the proposed mechanisms.

We believe that these revisions strengthen the rigor, transparency, and overall quality of the manuscript. We respectfully submit this revised version for your consideration and are happy to address any further questions.

Reviewer #1:

The manuscript by Villafuerte et al. investigates how naturally associated bacteria modulate facultative vivipary (internal egg hatching) in Caenorhabditis elegans. By leveraging the CeMbio microbiome resource together with well-established insulin/IGF-1 signaling mutants, the authors demonstrate that distinct commensal bacterial taxa induce internal hatching predominantly in late adulthood and engage different branches of the host insulin signaling network. The study is timely and addresses an important gap in our understanding of how natural microbiome members shape host reproductive strategies beyond pathogenic or starvation contexts. Overall, the work is well motivated, the experimental approaches are technically sound, and the conclusions are largely supported by the presented data. The integration of life-history traits with genetic dissection of insulin signaling is a particular strength. Nevertheless, several aspects of data presentation, clarity of interpretation, and mechanistic framing require further refinement to improve rigor and accessibility. Addressing these points would substantially strengthen the manuscript without necessitating major additional experimentation.

Response: We thank the reviewer for the thoughtful evaluation of our manuscript and for the positive assessment of the study. In response to the reviewer's suggestions, we have revised the manuscript to improve the clarity of data presentation, refine the interpretation of several results, and strengthen the mechanistic framing of the study. Specific revisions are detailed below in response to each comment.

Minor Comments
Comment 1. Statistical reporting
Please include exact p-values and sample sizes (number of worms analyzed) for all quantitative analyses, either in the main text, figure legends, or supplementary tables. In addition, a schematic model summarizing the proposed strain-specific engagement of insulin signaling branches would help readers grasp the key mechanistic conclusions.

Response 1.1: We thank the reviewer for this helpful suggestion. In the revised manuscript, we now report the exact p-values and sample sizes (number of worms analyzed) for all quantitative analyses. These details have been added to the corresponding figure legends and summarized in an updated supplementary table (Tables S1–S9) to ensure transparency of the statistical analyses.

In addition, as suggested by the reviewer, we have included a schematic model summarizing the proposed strain-specific engagement of host insulin signaling pathways. This model is presented as a new Figure 2A in the revised manuscript to help clarify the key mechanistic conclusions of the study.

Added text (Page 10 lines 308–323):

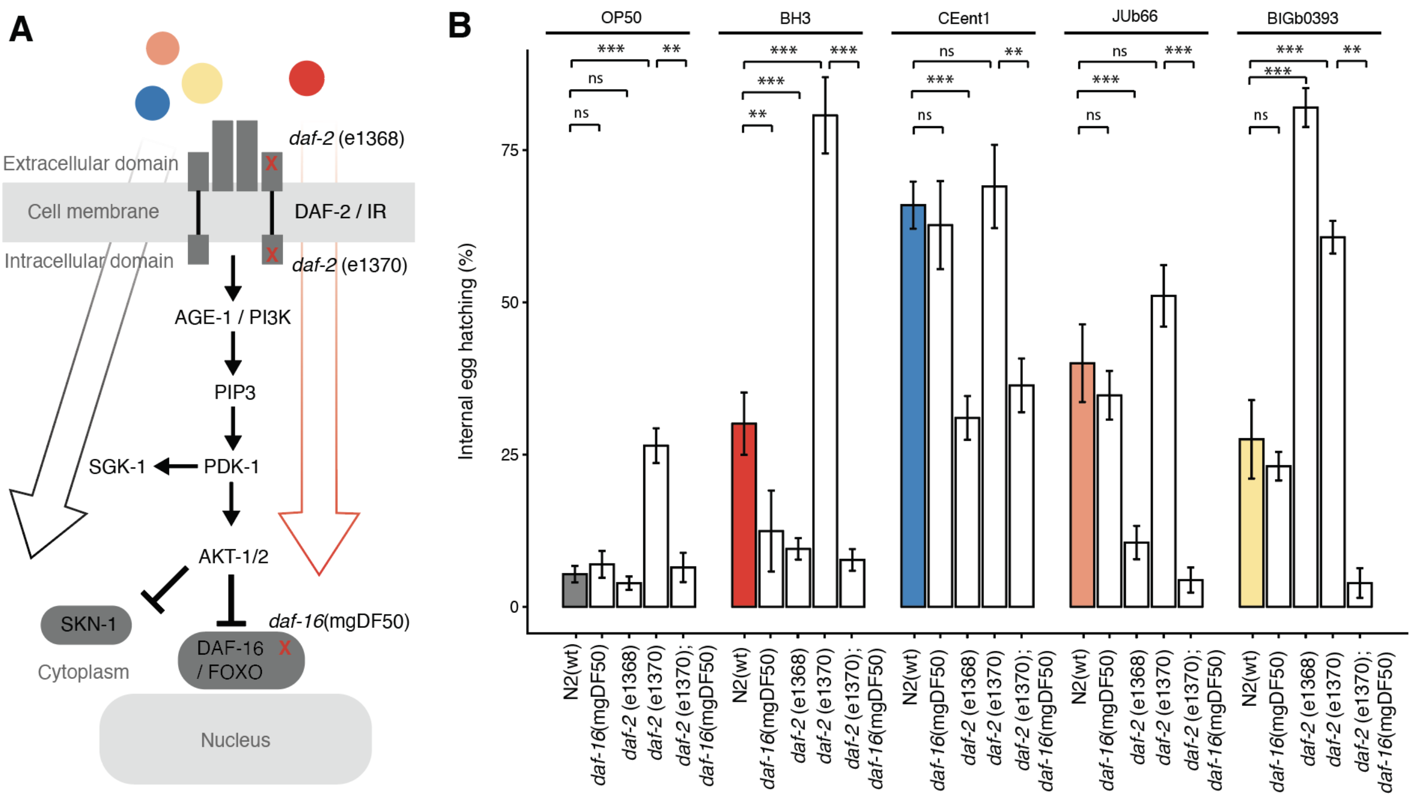

Figure 2. Interactions of focal strains with host insulin signaling. (A) Schematic of the C. elegans insulin/IGF-1 signaling pathway and its modulation by focal bacterial strains. Core components include DAF-2, AGE-1/PI3K, PDK-1, AKT-1/2, SGK-1, and the transcription factors DAF-16/FOXO and SKN-1/NRF2. Colored circles in the extracellular space represent bacterial-derived metabolites or signals from four focal strains. Loss of function mutation alleles (daf-2(e1368), daf-2(e1370), daf-16(mgDf50)) are indicated at their respective pathway positions. Shaded arrows denote proposed pathway branches engaged by bacterial strains based on genetic dependence observed in this study. (B) Internal egg hatching percentages of C. elegans insulin signaling mutants (daf-16(mgDf50), daf-2(e1368), daf-2(e1370), and daf-2(e1370);daf-16(mgDf50) double mutants) were quantified after exposure to focal bacterial strains. Data are shown as percentage of worms with internal hatching (mean ± SE, *adj p ≤ 0.05, ** adj p ≤ 0.01, *** adj p ≤ 0.001, N = 11–16 replicates per bacterial strain,

N = 25 to 30 individual animals per replicate). Exact statistical results are provided in Supplementary Tables S7–S9: S7 (summary statistics), S8 (Dunnett-adjusted comparisons of bacterial effects within genotype), and S9 (Dunnett-adjusted comparisons of genotype effects within bacterial condition).

Comment 2. Terminology clarification
Briefly define "facultative vivipary" at its first occurrence for readers who may not be specialists in C. elegans reproductive biology.

Response 1.2: We thank the reviewer for this helpful suggestion. We have now included a brief definition of “facultative vivipary” at its first occurrence in the Introduction.

Added text (Page 2 lines 58–60): This process, termed facultative vivipary, refers to the retention and internal hatching of embryos within the parent under specific physiological or environmental conditions.

Comment 3. Figure organization
For improved clarity and conciseness, consider combining Figure 2A and 2B into a single panel, and grouping Figure 2C, 2D, and 2E into a unified multi-panel figure with consistent formatting.

Response 1.3: thank the reviewer for this helpful suggestion. We reorganized Figure 2 into a unified multi-panel layout grouped by bacterial strain to facilitate comparison across *C. elegans* mutants, with consistent formatting and statistical annotations (See added text in Response 1.1). A supplementary figure (Figure S2) grouped by genotype has also been included to highlight bacterial effects across host genetic backgrounds.

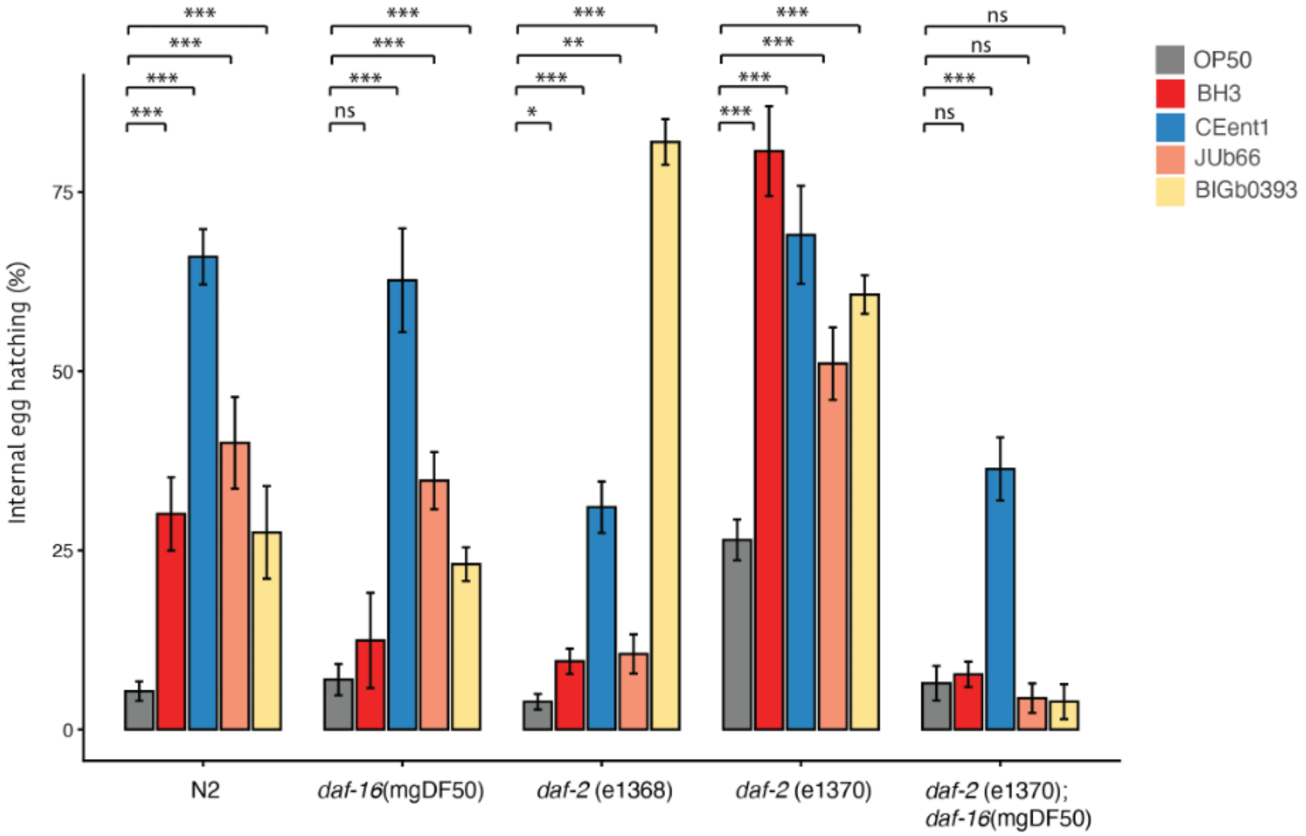

Figure S2. Internal hatching percentages of across bacterial conditions grouped by *C. elegans* genotype: N2, *daf-16*(dfmg50), *daf-2*(e1368), *daf-2*(e1370), and *daf-2*(e1370); *daf-16*(dfmg50). Data are shown as percentage of worms with internal hatching (mean ± SE). Statistical analyses were performed using GLMMs

with Dunnett-adjusted comparisons versus OP50 within each genotype. Exact statistics are provided in Supplementary Table S8.

Comment 4. Reference needed
Line 43: "leading to larvae hatching within the body that often results in premature maternal death."
Please provide an appropriate reference supporting this statement, as it is a key biological premise.

Response 1.4: We thank the reviewer for the suggestion. In the revised manuscript, we have added citations to prior studies describing internal hatching and its consequence of premature maternal death in C. elegans: Chalife, M., and White, J. (1980). The nervous system. In The nematode Caenorhabditis elegans (Cold Spring Harbor Laboratory Press), pp. 337–391.

Chen, J., and Caswell-Chen, E. (2003). Why Caenorhabditis elegans adults sacrifice their bodies to progeny. Nematology 5, 641–645. https://doi.org/10.1163/156854103322683355.

Added text (Page 2 lines 58): a phenomenon first described in early studies of egg-laying behavior in the 1980s

Comment 5. Text clarity
Line 18: "The three γ-proteobacterial strains belong to γ-proteobacteria"
This sentence is redundant and grammatically incorrect. Please revise for clarity and precision.

Response 1.5: Thank you for pointing this out. We have therefore removed the redundant text "belong to γ-proteobacteria" from the manuscript.

Comment 6. Reference needed
Line 61: "with the ~59% early adulthood induction reported for pathogens."
Please cite the original study or studies reporting this level of pathogen-induced internal hatching in early adulthood.

Response 1.6: We appreciate the suggestion and added the citation: Mosser, T., Matic, I., and Leroy, M. (2011). Bacterium-induced internal egg hatching frequency is predictive of life span in Caenorhabditis elegans populations. Appl. Environ. Microbiol. 77, 8189–8192.

Added text (Page 3 lines 88): 59% early adulthood induction reported for pathogens

Reviewer #2:

This manuscript investigates the influence of a number of naturally co-occurring bacteria (mainly from the CeMbio resource) on internal hatching in the nematode C. elegans. The study finds that some bacterial strains induce internal hatching preferentially in late adulthood, similar to what has been found for some pathogenic bacteria. The authors also use daf-2 and daf-16 mutants to make inferences about the involvement of insulin signalling.
The manuscript has conceptual and statistical weaknesses that limit the strength and reliability of the conclusions. Overall, the study feels very preliminary and the results should be treated/interpreted accordingly.

Response: We thank the reviewer for their careful evaluation of our manuscript and for raising important concerns. In response, we have undertaken substantial revisions to improve both the rigor of the analyses and the clarity of interpretation. Specifically, we implemented a generalized linear mixed-effects modeling framework that accounts for biological replicate (plate) effects and includes bacterial strain, genotype, and their

interaction as fixed effects. We also incorporated appropriate multiple-comparison corrections using Dunnett-adjusted contrasts and expanded statistical reporting throughout the manuscript, including exact p-values, sample sizes, and detailed results provided in revised figure legends and new supplementary tables.
We respectfully disagree with the characterization of the work as preliminary. This study is based on a systematic analysis of multiple naturally associated bacterial strains from the CeMbio resource, combined with genetic dissection of host insulin/IGF-1 signaling using well-established daf-2 and daf-16 mutants. These approaches enable the identification of reproducible, strain-specific effects on internal hatching across the adult lifespan.
To further strengthen the presentation, we refined the conceptual framing of the study. We have added a schematic model (Figure 2A) summarizing the proposed strain-specific interactions with host insulin signaling pathways. We also moderated our interpretation to emphasize that our data demonstrate genetic dependence on pathway components rather than direct activation of pathway activity. In addition, we expanded the discussion to better contextualize potential contributions of nutritional effects and to clearly outline limitations and future directions.

Main points
1. Interpretation of daf-2 signalling
The authors conclude that different bacterial strains engage distinct components of the insulin signaling pathway, including "canonical" and "non-canonical" signaling. This conclusion is based exclusively on phenotypic analysis of daf-2 and daf-16 mutants. No additional evidence is provided, e.g. measurements of pathway activity, which are relatively easy to obtain using available reporters. Overall, the data demonstrate genetic dependence, but not pathway engagement.

Response 2.1: We thank the reviewer for this important point and agree that our genetic analyses demonstrate pathway dependence rather than direct measurement of insulin signaling activity. We have revised the manuscript to clarify that bacterial strains differentially require components of the insulin/IGF-1 pathway (e.g., daf-2, daf-16) to modulate internal hatching, and have removed language implying direct engagement of distinct signaling branches. We now also note that direct measurements of pathway activity (daf-16 transcriptional or translational reporter) represent an important direction for future work.

Added text (Page 4 lines 126): These results indicate that BH3-induced internal hatching acts in a DAF-16-dependent manner.

Added text (Page 6 lines 179–183): Importantly, our findings reflect pathway dependence inferred from genetic perturbations and do not constitute direct measurements of signaling activity. Direct readouts of pathway activity, such as transcriptional or subcellular localization reporters for DAF-16, will be essential to determine how microbial signals are integrated within IIS to regulate reproductive outcomes.

2. Nutrient effects of bacteria
The bacteria exert their effects also / primarily as nutrition. This aspect is not or insufficiently discussed. The simplest interpretation is that bacteria inducing internal hatching represent low-quality or mildly pathogenic food sources (as further suggested by the reproductive output assays). It would have been interesting to test this specifically, e.g. by assessing whether bacteria that induce internal hatching also induce mild starvation. For example, see the recent paper, which used lipid stains to look at that.
Braun, N. J., and D. E. Mor (2025). Gut microbiome species Levilactobacillus brevis regulates reproductive fitness in C. elegans. Curr. Res. Microb. Sci. 9: 100471.
https://pubmed.ncbi.nlm.nih.gov/41018064/

Response 2.2: We thank the reviewer for this insightful comment and for highlighting the potential role of bacterial nutritional quality. We agree that differences in food quality or mild pathogenicity could contribute to

the observed phenotypes and have expanded the Discussion accordingly. Prior characterization of these strains showed accelerated developmental rates relative to OP50 (Dirksen et al 2020, G3), suggesting that they do not impose overt nutrient limitation during development. Consistent with this, we observed only modest reductions in total progeny, arguing against substantial nutrient deprivation during early adulthood. Together, these observations indicate that worms are unlikely to experience pronounced starvation during development and early reproductive stages under these conditions.

However, as host–microbe interactions are dynamic across the lifespan, reduced food quality or mild pathogenic effects may emerge later in adulthood (after day 4), potentially contributing to increased internal hatching. We have revised the discussion to explicitly consider this possibility and to note that nutritional status may be integrated through insulin signaling pathways to influence this phenotype. We also cited Braun and Mor (2025) and highlight lipid-based assays as an important direction for future work.

Added text (Page 6 lines 183–188): In addition, given the dynamic nature of host–microbe interactions across the lifespan, bacterial effects may shift over time. Although these strains support accelerated development relative to OP50 and robust reproduction, reduced nutritional quality or mild pathogenic effects may emerge later in adulthood after day 4. Direct metabolic readouts such as lipid staining during late adulthood will be needed to determine whether nutritional effects contribute to internal hatching observed.

3. Experimental design
The experimental design and statistical analyses are of insufficient quality. It is unclear whether all experiments that were compared together were actually performed in parallel. More importantly, it is unclear how ANOVAs were performed-no detailed statistical test results (degrees of freedom, F values, etc.) are provided. Apparently, plate/replicate effects were not taken into account, which is problematic/incorrect. Similarly, figures do not display such replicate variation. Crucially, bacteria were all pairwise compared to OP50, apparently without correcting for multiple comparisons. This is not transparent.
Figure 2: It is unclear which comparisons between genotypes/treatments were statistically tested and how.

Response 2.3: We thank the reviewer for the evaluation of the experimental design and statistical analyses. We have made the following revisions to improve clarity and statistical rigor.

1. We clarified in the Methods that all comparisons were performed using experiments conducted in parallel under matched conditions. We also specify that data in Figures 1A, 1B, 1F (internal hatching percentages for focal strains and egg-laying rates), and 2B were compiled from two independent experiments.
2. We revised the statistical framework for internal hatching analyses by applying generalized linear mixed-effects models with a binomial error structure. In these models, bacterial strain, genotype, and their interaction are included as fixed effects, and biological replicate (plate) is included as a random effect to account for non-independence among worms within plates.We also addressed multiple comparison concerns by applying Dunnett-adjusted contrasts for comparisons between bacterial strains and the OP50 control.
3. We expanded statistical reporting throughout the manuscript, including test statistics, adjusted p-values, sample sizes, and degrees of freedom where applicable. These exact test results are now provided in the figure legends and compiled in Supplementary Tables S1–S9.
4. For Figure 2, we clarified in both the figure legend and Methods the statistical comparisons were performed. Specifically, we tested for a bacteria × genotype interaction using likelihood ratio tests, followed by Dunnett-adjusted post hoc comparisons within each genotype and genotype comparisons within each bacterial condition.

Added text (Page 8 lines 244–258): All analyses were performed in R (v4.3.0) using dplyr, ggplot2, lme4, and survival packages. Internal hatching percentages was calculated as the proportion of worms exhibiting internal hatching relative to the total number of worms per plate and is presented as mean ± SE. These data were modeled as a binomial response using generalized linear mixed-effects models (GLMMs) implemented in the lme4 package, with bacterial strain and genotype included as fixed effects and biological replicate (plate) included as a random effect to account for non-independence within plates. Post hoc comparisons between bacterial strains and the OP50 control were performed using estimated marginal means with Dunnett-adjusted contrasts in the emmeans package. The significance of the bacteria × genotype interaction was assessed using likelihood ratio tests by comparing models with and without the interaction term. Total reproductive output was analyzed using one-way ANOVA followed by Tukey's HSD post hoc test. Daily reproductive output and egg laying rates were analyzed using mixed-effects models with bacterial strain, day, and their interaction as fixed effects and biological replicate included as a random effect. Post hoc comparisons were performed using estimated marginal means with Dunnett-adjusted contrasts.

Added text (Page 9 lines 273–296):
Figure 1. Natural commensal bacteria induce internal hatching and impact on lifespan and reproduction. (A) Mean percentage ± SE of C. elegans N2 that underwent internal egg hatching during late adulthood (Days 4–7) when grown on 14 bacterial strains in monoculture on NGM plates at 20 °C. Statistical significance was determined by comparison of natural bacterial strains to E. coli OP50 with linear mixed model with Dunnett-adjusted post hoc comparisons (*adj $p \leq 0.05$, ** adj $p \leq 0.01$, *** adj $p \leq 0.001$, N = 5–16 replicates per bacterial strain, N = 30 to 32 individual animals per replicate; see Supplementary Table S1 for details). (B) Cumulative daily percentage of worms that underwent internal hatching over adulthood. Points represent mean ± SE across biological replicates (N = 10–16 replicates per bacterial strain from two independent experiments, N = 30 to 32 individuals per replicate; see Supplementary Table S2 for details). (C) Kaplan–Meier survival curves showing daily survival probabilities of C. elegans adults. Internal hatching events were scored as censored worms and removed from survival calculations (N = 5 replicates per bacterial strain, N = 30 individuals per replicate; see Supplementary Table S3 for details). (D) Total number of progeny produced per worm (mean ± SE) over the reproductive period. Different letters above bars indicate statistically significant differences among bacterial treatments (one-way ANOVA followed by Tukey's HSD post hoc test, $p < 0.05$, N = 6 plate replicates for each bacterial strain; see Supplementary Table S4 for details). (E) Smoothed curves show the daily progeny produced per worm (mean ± SE) over the reproductive period. Significance on Day 1 adulthood is indicated for BH3 versus OP50. (N = 6 plate replicates for each bacterial strain; see Supplementary Table S5 for details) (F) Hourly egg-laying rates (mean ± SE) of adults grown on focal bacterial strains. Significance at 48 h post-L4 is indicated for four focal strains versus OP50. Data are compiled from two independent experiments (N = 14–30 individuals per time point per bacterial strain; see Supplementary Table S6 for details).

Added text (Page 10 lines 308–323):

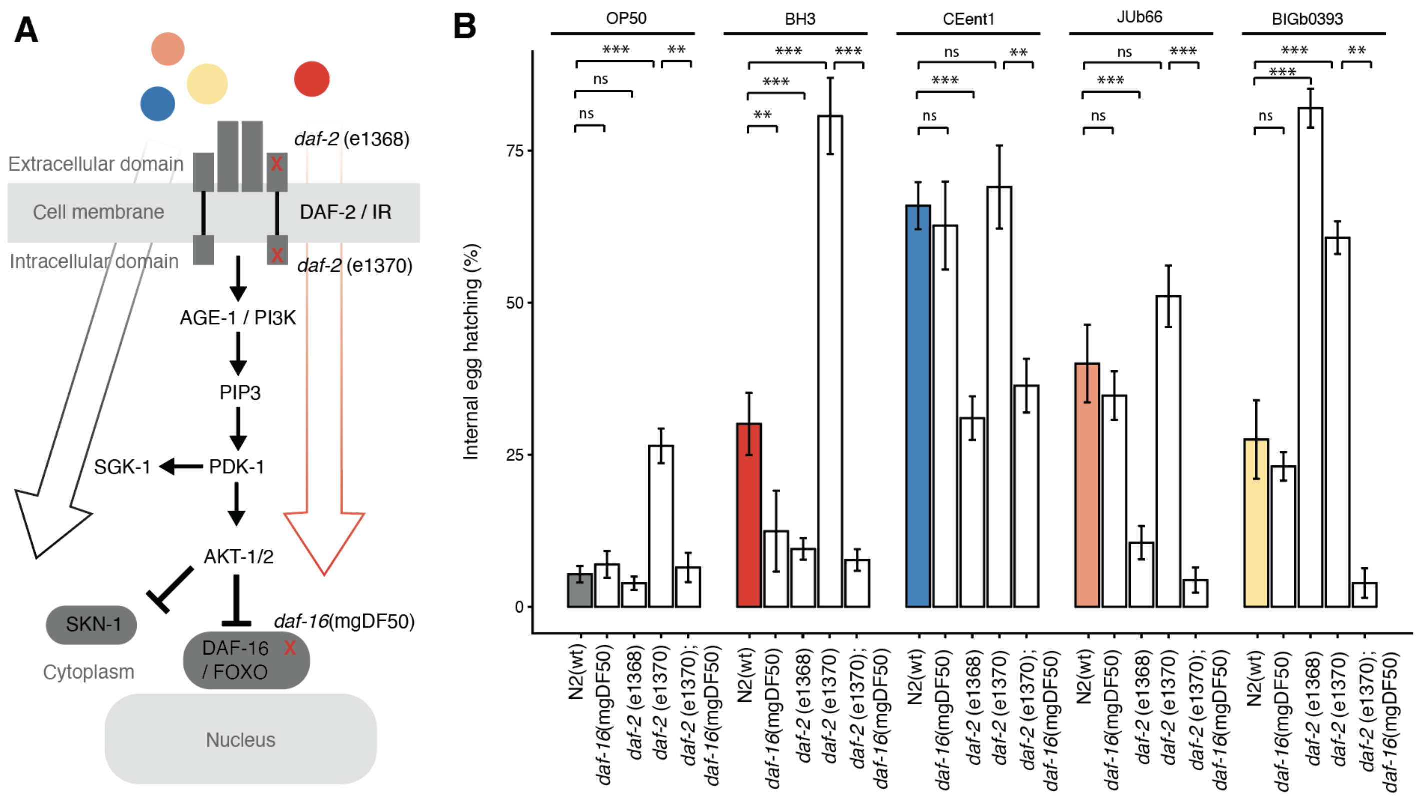

Figure 2. Interactions of focal strains with host insulin signaling. (A) Schematic of the C. elegans insulin/IGF-1 signaling pathway and its modulation by focal bacterial strains. Core components include DAF-2, AGE-1/PI3K, PDK-1, AKT-1/2, SGK-1, and the transcription factors DAF-16/FOXO and SKN-1/NRF2. Colored circles in the extracellular space represent bacterial-derived metabolites or signals from four focal strains. Loss of function mutation alleles (daf-2(e1368), daf-2(e1370), daf-16(mgDf50)) are indicated at their respective pathway positions. Shaded arrows denote proposed pathway branches influenced by bacterial strains based on genetic dependence observed in this study. (B) Internal egg hatching percentages of C. elegans insulin signaling mutants (daf-16(mgDf50), daf-2(e1368), daf-2(e1370), and daf-2(e1370);daf-16(mgDf50) double mutants) were quantified after exposure to focal bacterial strains. Data are shown as percentage of worms with internal hatching (mean ± SE, *adj $p \leq 0.05$, ** adj $p \leq 0.01$, *** adj $p \leq 0.001$, N = 11–16 replicates per bacterial strain from two independent trials, N = 25 to 30 individual animals per replicate). Exact statistical results are provided in Supplementary Tables S7–S9: S7 (summary statistics), S8 (Dunnett-adjusted comparisons of bacterial effects within genotype), and S9 (Dunnett-adjusted comparisons of genotype effects within bacterial condition).

Other comments

Abstract: It is unclear what the reported percentages refer to: "significantly increased the incidence of internal hatching (28-57%) during late adulthood."

Percent of individuals? Percent of populations? Increase relative to what baseline (0%, OP50, early adulthood)?

Response 2.4: We thank the reviewer for highlighting this lack of clarity. We have revised the text to specify that the reported percentages represent the proportion of individual worms exhibiting internal hatching relative to the total number of worms per plate within each bacterial condition during late adulthood. We have also clarified that these values are absolute (raw) measurements and are not expressed relative to the standard laboratory food source (E. coli OP50).

Added text (Page 1 lines 13–16): Among the bacterial strains tested, four strains (Ochrobactrum BH3, Lelliottia JUb66, Pantoea BIGb0393, and Enterobacter CEent1) induced high levels of internal hatching (28–57%) in C. elegans during late adulthood, compared to <5% in animals grown on E. coli OP50.

Lines 44-45:
"This facultative vivipary represents a plastic life-history trait that balances maternal survival and offspring fitness."
Comment: Reference 1 seems inappropriate here. Also, the term "stress-adaptive reproductive strategy" is unusual. Perhaps "a presumptive adaptive strategy that enhances survival under stressful conditions."

Response 2.5: We thank the reviewer for this helpful comment. We agree that the original reference was not the most appropriate to support this statement and have replaced it with more relevant literature. We have also revised the wording to improve clarity as suggested.

Added text (Page 2 lines 62–63): and may function as a presumptive adaptive strategy that enhances survival under stressful conditions in adulthood

Line 58: "OP50-fed worms"
Comment: Specify nematode genetic background (presumably wild-type N2). Also specify developmental stage at feeding (L1/egg or L4). Although mentioned elsewhere, it would be helpful to state it here.

Response 2.6: We thank the reviewer for this helpful suggestion. We have revised the text to specify that worms are wild-type N2 and to indicate the synchronized L1 larvae at which animals were grown to bacterial conditions.

Added text (Page 3 lines 85): Wild-type N2 worms fed OP50 from the L1 stage

Line 57: Ochrobactrum pituitosa (BH3)
Comment: Why was this species chosen?

Response 2.7: We thank the reviewer for this question. Ochrobactrum pituitosa (BH3) is not part of the CeMbio resource but is a naturally associated strain from wild C. elegans and a member of the BIGbiome community. It was included based on prior work linking its gut colonization to modulation of insulin signaling (Zhang et al 2021, Current Biology). We therefore included BH3 to assess its effects on internal hatching and to provide comparison with the CeMbio Ochrobactrum strain MYb71 from the same genus.

Added text (Page 3 lines 81–84): In addition, *Ochrobactrum pituitosa* (BH3), a naturally associated strain isolated from wild *C. elegans* and a member of the BIGbiome community, was included based on a previous study linking its colonization to host insulin signaling, and to compare with the CeMbio Ochrobactrum strain MYb71 from the same genus.

Line 61: Missing citation.

Response 2.8: We thank the reviewer for the suggestion and it is also noted by Reviewer 1. We have now added the appropriate citation. Mosser, T., Matic, I., and Leroy, M. (2011). Bacterium-induced internal egg hatching frequency is predictive of life span in Caenorhabditis elegans populations. Appl. Environ. Microbiol. 77, 8189–8192.

Lines 75-77:
"This extended reproductive window occurs at the expense of total fecundity and lifespan."

Comment: This should be reworded to avoid unwarranted causal interpretation. Why must this represent a trade-off?

Response 2.9: We thank the reviewer for the suggestion and agree that the original wording implied a causal trade-off that is not demonstrated by our data. We have revised the text to state that an extended reproductive window is associated with reduced total fecundity and lifespan, so to avoid this interpretation about causation.

Added text (Page 4 lines 107–109): This extended reproductive window is generally associated with reduced total fecundity and lifespan. BH3 represents an exception, as it enhances both lifespan and the duration of reproductive window.

Re: Spectrum04034-25R2 (**Natural commensals induce internal egg hatching in *C. elegans***)

Dear Dr. Fan Zhang:

Your manuscript has been accepted, and I am forwarding it to the ASM production staff for publication. Your paper will first be checked to make sure all elements meet the technical requirements. ASM staff will contact you if anything needs to be revised before copyediting and production can begin. Otherwise, you will be notified when your proofs are ready to be viewed. Please note that there are several small issues raised by Reviewer #2 that can be addressed during proofing - I ask that you make those changes at that time.

Data Availability: ASM policy requires that data be available to the public upon online posting of the article, so please verify all links to sequence records, if present, and make sure that each number retrieves the full record of the data. If a new accession number is not linked or a link is broken, provide production staff with the correct URL for the record. If the accession numbers for new data are not publicly accessible before the expected online posting of the article, publication may be delayed; please contact ASM production staff immediately with the expected release date.

Publication Fees: For information on publication fees and which article types have charges, please visit our website. We have partnered with Copyright Clearance Center (CCC) to collect author charges. If fees apply to your paper, you will receive a message from no-reply@copyright.com with further instructions. For questions related to paying charges through RightsLink, please contact CCC at ASM_Support@copyright.com or toll free at +1-877-622-5543. CCC makes every attempt to respond to all emails within 24 hours.

ASM Membership: Corresponding authors may join or renew ASM membership to obtain discounts on publication fees. Need to upgrade your membership level? Please contact Customer Service at Service@asmusa.org.

Embargo Policy: A press release may be issued as soon as the manuscript is posted on the Spectrum Latest Articles webpage. The corresponding author will receive an email with the subject line "ASM Journals Author Services Notification" when the article is available online.

The ASM Journals program strives for constant improvement in our submission and publication process. Please tell us how we can improve your experience by taking this quick Author Survey.

Thank you for submitting your paper to Spectrum.

Sincerely,
John Chaston
Editor
Microbiology Spectrum

Reviewer #2 (Comments for the Author):

The revised manuscript improves statistical rigor, clarity, and interpretation, adressing major reviewer concerns while strengthening the overall presentation and mechanistic framing, which is tuned down. Remaining issues are minor and mainly involve tightening language, improving flow, and ensuring consistent terminology.
- use viviparity (not vivipary)
- with regard to Added text (Page 2 lines 62-63): and may function as a presumptive adaptive strategy that enhances survival under stressful conditions in adulthood
  this has been previously shown experimentally, and could be cited here:
Mignerot L., C. Gimond, L. Bolelli, C. Bouleau, A. Sandjak, et al., 2024 Natural variation in the Caenorhabditis elegans egg-laying circuit modulates an intergenerational fitness trade-off. Elife 12. https://doi.org/10.7554/eLife.88253

Reviewer #3 (Comments for the Author):

The revised manuscript appears to address the major concerns raised in previous review, satisfactorily.
